# Surface Performance of Nano-CrN/TiN Multi-Layered Coating on the Surface of Ti Alloy

**DOI:** 10.3390/ma16247707

**Published:** 2023-12-18

**Authors:** Jun Feng, Zhiyong Shi, Yingchao Zhao, Jun Wang, Xudong Yang, Mingchun Zhao

**Affiliations:** 1 College of Mechanical Engineering, University of South China, Hengyang 421001, China; fengjun@usc.edu.cn (J.F.); 2002000222@usc.edu.cn (J.W.); 20212006210344@stu.usc.edu.cn (X.Y.); 2 School of Materials Science and Engineering, Central South University, Changsha 410083, China; 213111066@csu.edu.cn; 3 Hunan Advanced Manufacturing Engineering Technology Research Center of High-Wear-Resistant Alloy Materials, Hunan Lifang Rolling Roll Co., Ltd., Hengyang 421600, China

**Keywords:** surface coating, nano-multi-layered structure, wear, high-temperature oxidation

## Abstract

Surface coating has been widely used to ameliorate the surface properties of Ti alloys. In this study, high-power pulsed magnetically controlled sputtering technology was used to successfully prepare a nano-CrN/TiN multi-layered coating on the surface of a TC4 Ti alloy. The surface of the obtained coating was uniform, dense, and free of obvious defects. With the decrease in modulation period, the optimal growth of the nano-CrN/TiN multi-layered coating was changed from a (220) crystal surface to (111) and a (200) crystal surface. Compared to the single-layered CrN or TiN coating, the nano-multi-layered coating had higher hardness and lower wear rate. Furthermore, the hardness and the wear resistance increased with the decrease in the modulation period. This presented an optimal modulation period of 6 nm. Meanwhile, the resistance of the obtained coating to high-temperature oxidation at 800 °C was also significantly improved.

## 1. Introduction

TC_4_-Ti (Ti_6_Al_4_V) alloys have been widely used in the aerospace field due to their high specific strength and good thermal stability [1,2]. However, Ti alloys have the disadvantages of low surface hardness, poor high-temperature oxidation resistance, a high friction coefficient, and easy adhesion wear, which limit their commercial application [3,4,5]. Kermanpur et al. [6] reported that the occurrence of failure was related to the low wear resistance of the Ti alloy blade’s root. Surface coating has been widely used to ameliorate the problems mentioned above [7,8,9,10]. At present, two common thin coatings, i.e., TiN coating and CrN coating, are preferred as the surface reinforcement layer for applications in surface protection of Ti alloys because they can meet some special performance demands [11,12,13]. By comparing these two coatings, the TiN coating can provide higher coating hardness and frictional wear resistance [14], while the CrN coating can provide higher toughness and oxidation resistance. Harish et al. [15] reported that the TiN coating had higher hardness than the CrN coating but provided insufficient heat resistance and was prone to oxidation. Pei et al. [16] reported that the rapid oxidation of the TiN coating occurred at 873 K, resulting in coating degradation and failure. Chim et al. [17] reported that the TiN coating and the CrN coating suffered severe oxidation at 973 K; however, comparatively speaking, the CrN coating had better high-temperature oxidation resistance than the TiN coating. Upadhyay et al. [18] reported that the high-temperature oxidation resistance and the toughness of the CrN were higher than the TiN coating.

On the other hand, the pursuit of material properties is endless. Nano-multi-layer coatings have been substantiated to have better mechanical properties, high-temperature oxidation resistance, and tribology properties than single-layer coatings [19]. In the context of suitable interface and performance matching of Ti alloy substrates, combining a TiN coating with a CrN coating to produce nano-TiN/CrN multi-layer coatings should offer an approach to meet the increasing performance demands of Ti alloys due to their inheritance of the advantageous properties of both the TiN coating and the CrN coating. In particular, the TiN coating and the CrN coating have a low lattice mismatch and can even form a superlattice structure nano-TiN/CrN multi-layer coating [20]. This superlattice multi-layer structured coating was developed from two kinds of coatings with well-matched lattice, which alternately grew in thin layers of several nanometers to tens of nanometers and maintained strict periodicity. As reported, the superlattice multi-layer structured coating had the advantages of reducing internal stress and increasing toughness, hardness, and base adhesion [20].

Various methods, including magnetically controlled sputtering and arc ion plating technology, have been explored to prepare nano-TiN/CrN multi-layer coatings [21,22,23]. The main problem with using these traditional preparation methods was insufficient ionization rate, which easily generated large particles, resulting in an uneven and defective coating and, consequently, damaged its performance. In contrast, high-power magnetically controlled sputtering technology can improve the ionization rate, which does not form large particles on the obtained coatings layer, makes the coating dense, and has fewer defects, thus attracting more attention in the preparation of nano-multi-layer CrN/TiN coatings [24,25]. Paulitsch et al. [26] produced low-friction CrN/TiN multi-layer coatings using high-power impulse magnetron sputtering. Lin et al. [27] described that high-power pulse magnetron sputtering technology had wide application prospects in the preparation of CrN/TiN multi-layer coatings. Nevertheless, the details of the preparation and the surface performance of nano-multi-layer CrN/TiN coatings via high-power magnetically controlled sputtering technology are still unclear and need further clarification, as are the details of the factors influencing the growth and, further, the performance of nano-multi-layer CrN/TiN coatings under different modulation periods.

In this work, high-power pulsed magnetically controlled sputtering technology was used to prepare nano-CrN/TiN nano-multi-layer coatings on the surface of a TC4 Ti alloy. The details of the preparation and the performance of the obtained nano-multi-layer CrN/TiN coating were studied by changing the modulation period and modulation ratio in the case of maintaining the thickness of the fixed coatings.

## 2. Experiments

**Figure 1:**
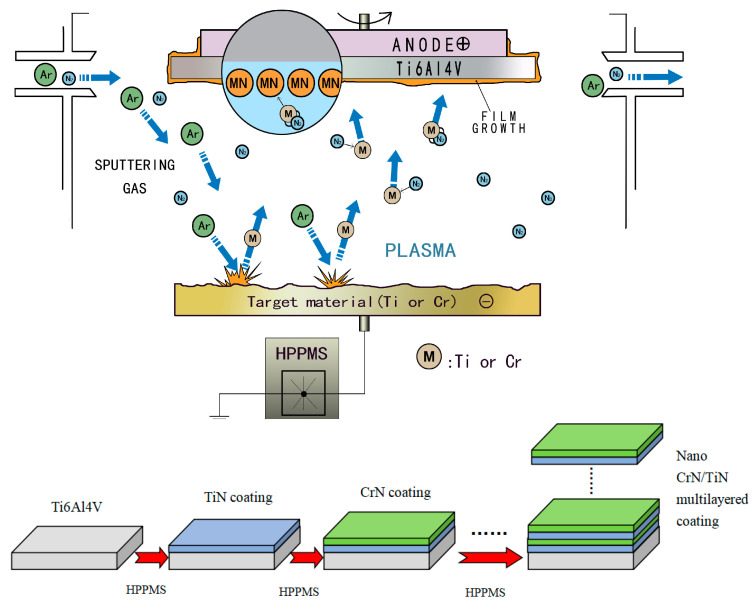
The preparation process of nano-CrN/TiN multi-layered coating.

A TC_4_-Ti (Ti_6_Al_4_V) alloy sheet with the dimensions 10 mm × 10 mm × 5 mm was used as the substrate material. Figure 1 shows the preparation process of the nano-CrN/TiN multi-layered coating. The coating was deposited by high-power pulse magnetically controlled sputtering equipment (Southwest Institute of Physics of the Nuclear Industry, Chengdu, China). Pure Ti (with a purity of ~99.99%) and pure Cr (with a purity of ~99.99%) were used as the Ti target and Cr target, respectively. Argon gas (purity 99.99%) was used as the sputtering gas, and nitrogen gas (purity 99.99%) was used as the reaction gas. The base specimens were ultrasonically cleaned for 10 min with acetone and aqueous ethanol, dried with nitrogen gas, and placed in the vacuum chamber cavity. During the deposition process of the coating, the working pressure was 0.5 Pa, the target–substrate interval space was 20 cm, the pulse bias was 240 V, the frequency was 300 Hz, the duty cycle was 8%, and the deposition temperature was 330 °C. The TiN coating or the CrN coating was obtained by rotating the substrate directly against the Ti target or Cr target, respectively. In such experimental conditions, the TiN coating or the CrN coating was deposited for 30 min, and their growth rates were calculated as 0.45 nm/s and 0.3 nm/s, respectively, according to the thickness of the obtained corresponding coating obtained.

The morphology of the coating was characterized by a scanning electron microscope (SEM, Jeol JSM 5900 LV, Tokyo, Japan), a transmission electron microscope (TEM, Tecnai F20, Eindhoven, The Netherlands), and X-ray diffraction (XRD) with Cu Ka radiation at 40 kV and 30 mA, using a scan rate of 0.05°/s in the 2θ range of 25–90°. The hardness was measured via a Vickers micro-hardness tester (YZHV-1000C, Shanghai, China) equipped with a Knoop indenter and operated at an applied load of 10 gf. The friction-and-wear test was conducted by an SRV reciprocating sliding wear tester (Optimol SRV, Jena, Germany) using Al_2_O_3_ grinding balls (purity of 99.5%) with diameters of 3 mm. The oxidation test was conducted in an electric furnace in static air at 1073 K for 4 h, followed by furnace cooling to room temperature. After the oxidation test, the distribution of element contents with coating depth was analyzed by auger electron spectroscopy (AES, PHI-700, Chigasaki, Japan).

## 3. Results and Discussion

### 3.1. Construction

The performance of the nano-CrN/TiN multi-layered coating is associated with the modulated period (Λ), which refers to the sum of the thicknesses of adjacent TiN coating and CrN coating in multi-layer coating. In this study, Λ is calculated by Equation (1), and the total thickness of the coating, D*_total_*, is calculated by Equation (2).

Λ = D_1_ + D_2_ (1)

D*_total_* = N Λ = N (D_1_ + D_2_) (2)

where D_1_ is the thickness of the single-layered TiN coating (unit in nm), D_2_ is the thickness of the single-layered CrN coating (unit in nm), and N is the number of all the layers of both the single TiN coating and the CrN coating. Under the condition of the stable deposition rate of the TiN coating (0.45 nm/s) and the CrN coating (0.3 nm/s), the Λ values may be 6, 9, 15, and 30 nm by alternately adjusting their deposition time, which can also make the single-layer TiN coating twice as thick as the single-layer CrN coating, and finally, the thickness of the nano-CrN/TiN multi-layered coating was ~3 μm.

**Figure 2:**
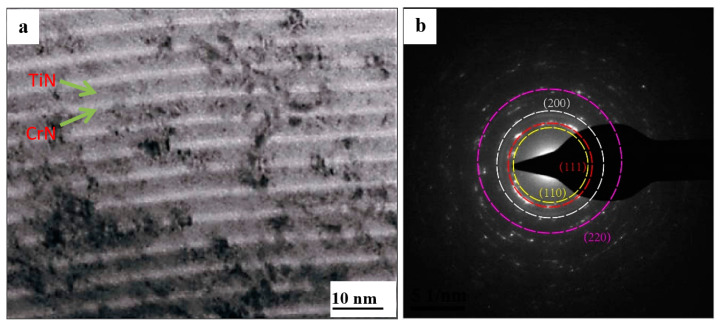
TEM image of a cross-section of the coating obtained at a modulation period of 6 nm. (**a**) TEM micrograph (**b**) selected area electron diffraction (SAED) pattern.

Figure 2 shows the typical TEM micrograph and the corresponding selected area electron diffraction (SAED) pattern of the cross-sectional side view of the coating obtained at a modulation period of 6 nm, which presents a multi-layered structure with alternating light and dark. The bright layer and the dark layer were a CrN layer and a TiN layer, respectively. The thickness of the bright layer was uniform, as was the thickness of the dark layer. Furthermore, the thickness of the dark layer (~20 nm) was approximately twice that of the bright layer (~10 nm). The SAED pattern showed a diffraction ring on (111), (200), and (220) crystal planes with continuous bright circular characteristics, further indicating the formation of a nanometer polycrystalline structure [28,29,30]. The crystal structure parameters of TiN and CrN were similar, so their diffraction rings almost coincided.

**Figure 3:**
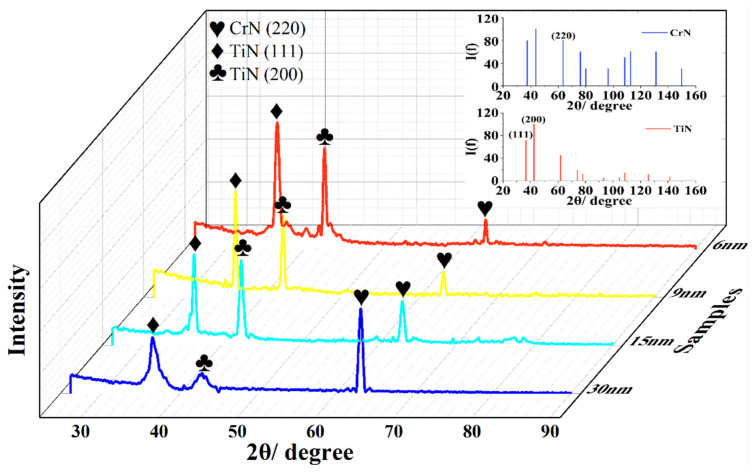
XRD results of the nano-CrN/TiN multi-layered coating.

Figure 3 shows the XRD results of the obtained coatings at different modulation periods, in which the diffraction peak positions correspond to the CrN (PDF # 11-0065) and TiN (PDF # 38-1420) standard PDF cards, respectively. This presented three obvious diffraction peaks, which were located near CrN (220) (~61.8°), TiN (111) (~36.6°), and TiN (200) (~42.6°), respectively. At a modulation period of 6 nm, (111) and (200) were the preferred orientations. When the modulation period was increased, the preferred orientation was changed from (111) and (200) to (220). The change in the preferred orientation was related to the following mechanism. When the modulation period was low (for example, 6 nm), the CrN coating was in the early stage of growth, and the TiN coating (with the preferred orientation of (111) and (200)) acted as the growth template for the CrN coating [31,32,33]. When the modulation period was increased, the thickness of the CrN coating was increased, and accordingly, its initial growth mode changed, resulting in the change in the orientation. In addition, a suspected satellite peak (~41.8°) was located on the left side of the (200) diffraction peak at a modulation period of 6 nm. This implied the formation of a steep interface in the nano-multi-layered coating [34,35].

### 3.2. Hardness

**Figure 4:**
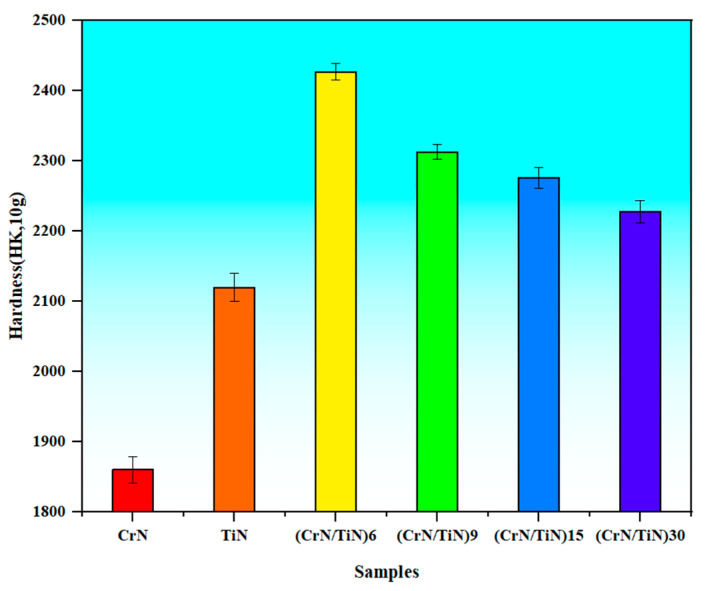
Knoop hardness of nano-CrN/TiN multi-layered coating, with a single-layered CrN coating and a single-layered TiN coating as a comparison.

Figure 4 shows the hardness values of the obtained coatings at different modulation periods. The hardness values of a single-layered CrN coating and a single-layered TiN coating were ~1860 and ~2120, respectively, which are also depicted in Figure 4 for a comparison. Compared to the single-layered CrN or TiN coating, the hardness values of the obtained multi-layered coatings were significantly increased. Furthermore, there was a change in the hardness value as the modulation period changed. When the modulation period was 30 nm, the hardness value was ~2228. With a decrease in the modulation period, the hardness value increased. When the modulation periods were 15, 9, and 6 nm, the hardness values were ~2276, ~2313, and ~2427, respectively. In particular, the decrease in the hardness value was very remarkable when the modulation period was decreased from 9 nm to 6 nm. As reported in [36], at a certain modulation period, a superlattice structure was produced in the nano-CrN/TiN multi-layered coating, resulting in ultra-hardening and, hence, effectively improving the hardness value of the coating. The hardening of the superlattice coating might be attributed to the significant difference in the shear modulus between the CrN (125 GPa) and the TiN (192 GPa), which caused the interlayer interface to hinder the dislocation movement [37,38,39]. Compared to the coatings in the present experiment, the modulation period of 6 nm was closer to the modulation period that might produce a superlattice structural coating, which has the advantages of increased hardness and base adhesion [20].

### 3.3. Wear

**Figure 5:**
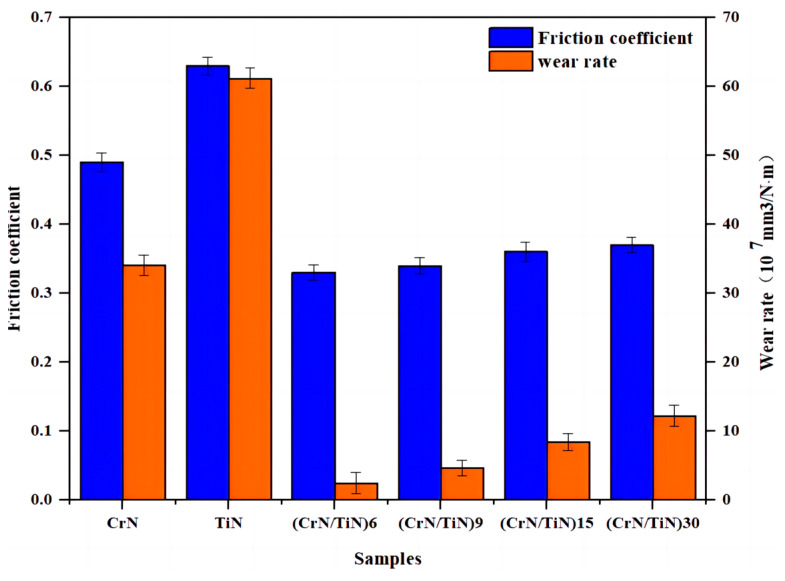
Wear rates and friction coefficients of coating vs. period, with a single-layered CrN coating and a single-layered TiN coating as a comparison.

Figure 5 shows the friction coefficients and the wear rates of the nano-multi-layered coating at different modulation periods. Those of a single-layered CrN coating and a single-layered TiN coating are also depicted in Figure 5 as a comparison. The friction coefficient and the wear rate of the single-layered TiN were ~0.63 and ~6.12 × 10^−6^ mm^3^/(N·m), and those of the single-layered CrN were ~0.49 and ~3.41 × 10^−6^ mm^3^/(N·m), respectively. In contrast, those of the nano-CrN/TiN multi-layered coatings were significantly decreased. Moreover, they decreased with the decrease in the modulation period. For example, at the modulation periods of 30 nm, 15 nm, 9 nm, and 6 nm, the friction coefficients were ~0.37, ~0.36, ~0.34, and ~0.33, respectively, and the wear rate was ~1.22 × 10^−6^ mm^3^/(N·m), ~8.41 × 10^−7^ mm^3^/(N·m), ~4.67 × 10^−7^ mm^3^/(N·m), and ~2.45 × 10^−7^ mm^3^/(N·m), respectively. Therefore, the nano-CrN/TiN multi-layered coating obtained at a modulation period of 6 nm (hereafter, the nano-CrN/TiN multi-layered coating −6 nm) had relatively minimal wear.

**Figure 6:**
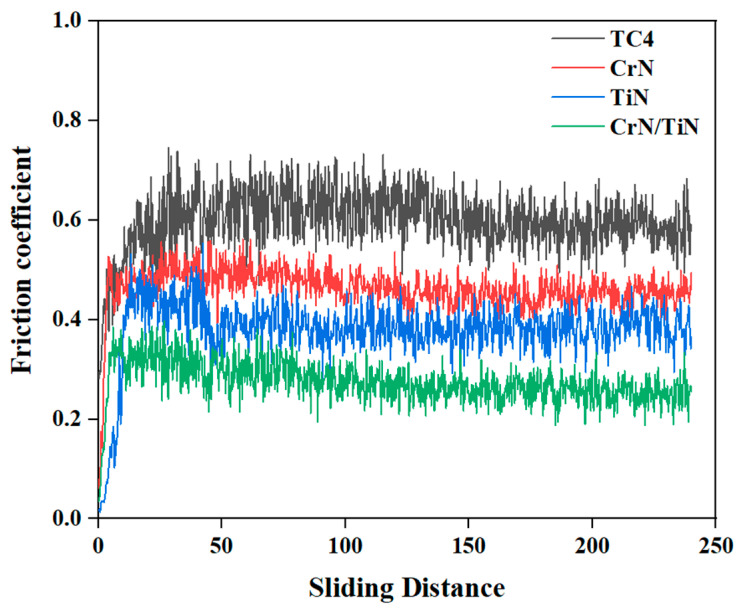
Friction curves of the TC4 Ti alloy substrate and the different coatings.

Figure 6 shows the friction coefficients of the TC4 Ti alloy substrate, the single-layered TiN coating, the single-layered CrN coating, and the nano-CrN/TiN multi-layered coating −6 nm. All measured curves had a minor fluctuations, which might be attributed to the contact material of the friction pair. The contact material used in the present wear test was Al_2_O_3_ grinding balls, which were wear-resistant metal ceramics and had much better wear resistance than the tested coating samples. In the wear test, plastic deformation, material adhesion, and material peeling occurred on the surface of the tested coating samples due to the combined effect of adhesive wear, fatigue wear, and abrasive wear. The process of material adhesion and detachment on the surface increased the tangential force, which led to the fluctuation of the friction coefficient. As shown in Figure 6, the friction coefficient of the TC4 Ti alloy substrate was ~0.63, that of the CrN coating was ~0.49, that of the TiN coating was ~0.43, and that of nano-CrN/TiN multi-layered coating −6 nm was ~0.37. Therefore, nano-CrN/TiN multi-layered coating −6 nm had a significantly lower friction coefficient than those aforementioned and, accordingly, had better friction and wear performance.

**Figure 7:**
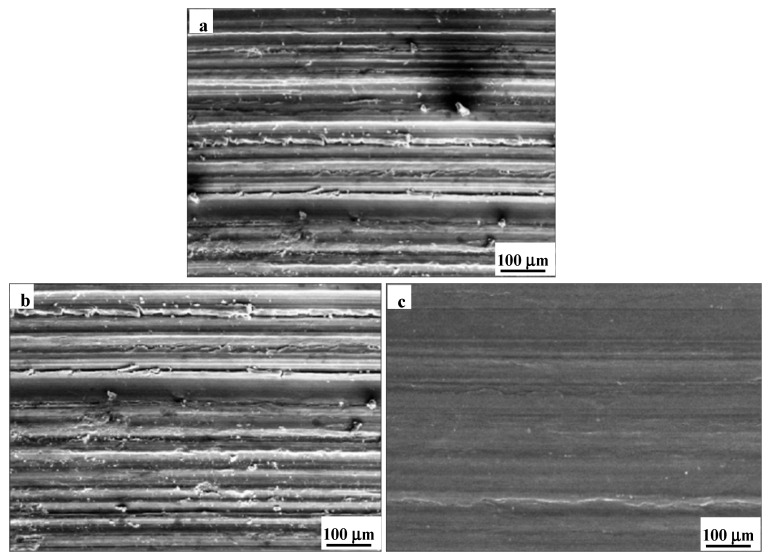
SEM images of wear surfaces of the different coatings: (**a**) TiN coating, (**b**) CrN coating, (**c**) nano-CrN/TiN multi-layered coating −6 nm.

Figure 7 shows the SEM images of the wear surface of the single-layered TiN coating, the single-layered CrN coating, and the nano-CrN/TiN multi-layered coating −6 nm after the friction and wear experiments. For the single-layered TiN coating (Figure 7a) and the single-layered CrN coating (Figure 7b), very obvious plow grooves appeared on the surface, parallel to the sliding direction, and some small wear debris were scattered nearby the plow grooves. In contrast, for the nano-CrN/TiN multi-layered coating −6 nm, shallow plow grooves appeared on the surface, with relatively flat and clean morphology characteristics (Figure 7c). After combining the data from the friction and wear experiments, it was found that plowing was the main material removal force during the present wear process. The single-layered TiN coating and the single-layered CrN coating mainly suffered from severe adhesive wear and abrasive wear, while the nano-CrN/TiN multi-layered coating −6 nm mainly suffered from slight adhesive wear and abrasive wear. Furthermore, the nano-CrN/TiN multi-layered coating −6 nm had relatively minimal wear. During the wear process, the coating samples were subjected to a force exceeding the load range and underwent plastic deformation from the beginning, causing wear in the early stage. With further wear, some debris were accumulated, which might have led to the change in the wear mechanism from adhesive wear to abrasive wear. This is because the coating surface was a three-dimensional porous structure [40,41], and the protrusions were worn and peeled off by the hard grinding ball. During the wearing process, a strain-hardened layer on the coating friction surface was formed by plastic deformation [42,43]. Therefore, the wear resistance largely relied on the coating itself. The obtained results substantiated that the nano-CrN/TiN multi-layered coating had good wear resistance.

### 3.4. High-Temperature Oxidation

**Figure 8:**
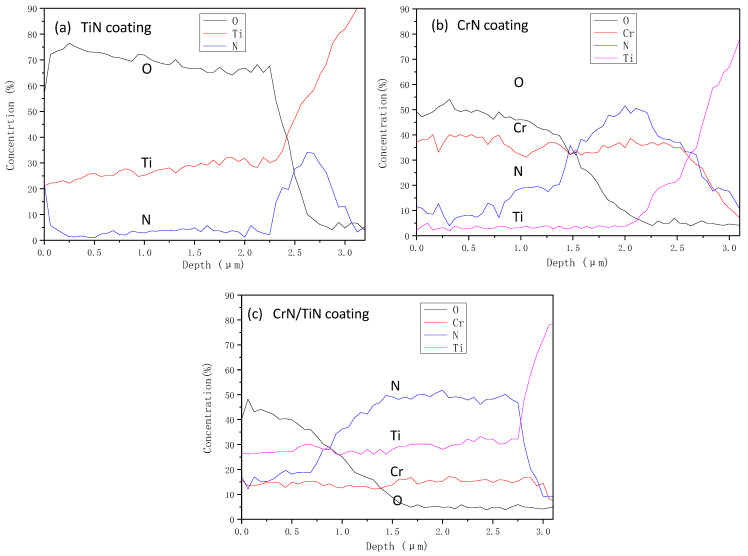
Plots of elemental distribution vs. distance for different coatings: (**a**) TiN coating, (**b**) CrN coating, (**c**) nano-CrN/TiN multi-layered coating −6 nm.

Figure 8 shows the AES (auger electron spectroscopy) results of the single-layered TiN coating, the single-layered CrN coating, and the nano-CrN/TiN multi-layered coating −6 nm after the oxidation test. Here, the oxygen content exceeding 20 wt.% was defined as high oxidation, and that less than 5 wt.% was defined as low oxidation.

For the single-layered TiN coating (Figure 8a), the results showed upper shelf O content (~70 wt.%) in the coating depth range of ≤2.25 μm, a gradual decrease in O content from the upper shelf to the lower shelf in the coating depth range of 2.25 μm~2.75 μm, and a lower shelf O content (~5 wt.%) in the coating depth range of ≥2.75 μm. The oxygen content of 20 wt.%, which corresponded to the high oxidation, was determined to be at the coating depth of 2.55 μm, while the oxygen content of 5 wt.%, which corresponded to low oxidation, was determined to be at the coating depth of 2.75 μm. Therefore, it could be concluded that a ~2.55 μm thick high-oxidation layer was formed, and the entire oxide layer was ~2.75 μm. According to the above results, the single-layered TiN coating with a thickness of ~3.0 μm formed a ~2.55 μm thick high-oxidation layer, indicating severe oxidation [44,45,46,47]. The resistance to high-temperature oxidation of the single-layered TiN was low.

For the single-layered CrN coating (Figure 8b), similar O content and coating depth curves were found, still having a gradual transition from an upper shelf O content to a lower shelf O content when increasing the coating depth. However, the upper shelf O content decreased to ~50 wt.%, and the O content gradually decreased from the upper shelf of ~50 wt.% to the lower shelf of ~5 wt.% in the coating depth range of 1 μm~2.25 μm. The oxygen content of 20 wt.% (the high oxidation) was determined to be at the coating depth of 1.74 μm, while the oxygen content of 5 wt.% (the low oxidation) was determined to be at the coating depth of 2.25 μm. The corresponding ~1.74 μm thick high-oxidation layer of was formed, and the entire oxidation layer was ~2.25 μm. Therefore, the single-layered CrN coating with a thickness of ~3.0 μm formed a ~1.74 μm high oxidation layer. Compared to the single-layered TiN, the resistance of the single-layered CrN to high-temperature oxidation was relatively improved, but not yet satisfactory.

For the nano-CrN/TiN multi-layered coating −6 nm (Figure 8c), there was no typical upper O content as above-mentioned, but the O content gradually decreased from the initial value of ~45 wt.% to the lower shelf of ~5 wt.% at the coating depth range of ~1.5 μm. The oxygen content of 20 wt.% (the high oxidation) was determined to be at the coating depth of 0.82 μm, while the oxygen content of 5 wt.% (the low oxidation) was determined to be at the coating depth of 1.5 μm. The corresponding ~0.82 μm thick high oxidation layer of was formed, and the entire oxidation layer was ~1.5 μm. Therefore, the nano-CrN/TiN multi-layered coating −6 nm with a thickness of ~3.0 μm formed a ~0.82 μm thick high-oxidation layer. Compared to the single-layered TiN coating and the single-layered CrN coating, the high oxidation layer thickness of the nano-CrN/TiN multi-layered coating was lower, hence its resistance to high-temperature oxidation was significantly better.

From the above results, the single-layered TiN coating and the single-layered CrN coating suffered from severe oxidation at 800 °C. The possible oxide phases (TiO_2_ and Cr_2_O_3_) can form at the surface, and the following reactions are possible:

2TiN + 2O_2_ = 2TiO_2_ + N_2_ (3)

4CrN + 3O_2_ = 2Cr_2_O_3_ + 2N_2_ (4)

Compared to the single-layered CrN coating, the single-layered TiN coating had more severe oxidation due to its low thermal stability. In contrast, the nano-CrN/TiN multi-layered coatings were generally relatively dense, and thereby, the combined effect of the dense TiO_2_ and Cr_2_O_3_ generated prevented further diffusion of oxygen. As a consequence, the nano-CrN/TiN multi-layered coating had a better resistance to high-temperature oxidation at 800 °C.

## 4. Conclusions

Nano-CrN/TiN multi-layer coatings were successfully prepared on the surface of a TC4 Ti alloy using high-power pulsed magnetically controlled sputtering technology. The details of the preparation and performance of the obtained nano-multi-layer CrN/TiN coating were studied by changing the modulation period and modulation ratio in the case of maintaining the thickness of the fixed coatings. This work provides a prospect for the development of nano-CrN/TiN multi-layer coatings to ameliorate the surface properties of Ti alloys. The main conclusions were obtained as follows:

1. A nano-CrN/TiN multi-layered coating was successfully prepared on the surface of a TC4 Ti-alloy using high-power pulse magnetically controlled sputtering technology. The coating was uniform, dense, and free of obvious defects and grew optimally on (111) and (200) crystal surfaces as the modulation period decreased.
2. Compared to the single-layered CrN coating and the single-layered TiN coating, the present nano-multi-layered coating had a higher hardness and reached the maximum hardness at the modulation period of 6 nm.
3. The wear rate of the nano-CrN/TiN multi-layered coating ranged from 1.22 × 10^−6^ to 2.45 × 10^−7^ mm^3^/(N·m), lower than the single-layered CrN and TiN coatings, and had a minimum of 2.45 × 10^−7^ mm^3^/(N·m) at a modulation period of 6 nm.
4. Compared to the single-layered CrN and TiN coatings, the nano-CrN/TiN multi-layered coating had better resistance to high-temperature oxidation at 800 °C.

## Data Availability

All data included in this study are available upon request by contact with the corresponding author.
